# An Ecological Inquiry on Young People’s Suicidal Ideation at University: Individual, Relational and Cultural Factors and Their Interactions

**DOI:** 10.3390/ejihpe14010005

**Published:** 2023-12-25

**Authors:** Silvana Mabel Nuñez Fadda, Hugo César Ciambelli Romero, Naiara Sophia Gradilla Lizardo, Jorge Alejandro Sánchez Castillón

**Affiliations:** Child and Youth Research Laboratory (LAIIF), Psychology Department, Campus Vallarta, Coast University Center, University of Guadalajara, Puerto Vallarta 48280, Mexico; hugo.ciambelli5903@academicos.udg.mx (H.C.C.R.); naiara.gradilla7789@alumnos.udg.mx (N.S.G.L.); jorge.scastillon@academicos.udg.mx (J.A.S.C.)

**Keywords:** suicidal ideation, university students, psychological distress, loneliness, family function, community support, virtual social media, gender

## Abstract

To understand the factors related to suicidal ideation in university students, we examined individual and microsystemic variables with an ecological model organizing bidirectional influences between different dimensions. Suicidal ideation, psychological distress, multidimensional self-concept, loneliness, community social support, life satisfaction, family functioning, bullying victimization, and problematic use of virtual social networks were measured in a sample of 376 Mexican university students (67% women) from 18 to 34 years of age (M = 20.8). Data were collected in March 2020, before mandatory confinement for COVID started in Mexico. Discriminant analyses showed that psychological distress, loneliness, self-concept, life satisfaction, family functioning, internet violence/rejection, and informal social support predicted belonging to high or no suicidal ideation groups with 88% accuracy. Only psychological distress and family self-concept predicted suicidal ideation in multilinear regression analyses. There were differences by gender in multilinear regression, with family self-concept significant for women and physical self-concept, but not family self-concept for boys. Conclusions: Early Suicide prevention in universities should include periodic screening of psychological distress, loneliness, and virtual social media use to identify students that need further evaluation and intervention. University-based strategies of mental health promotion that strengthen family relationships and the sense of community, including gender-sensitive orientation, could enhance the effect of protective factors.

## 1. Introduction

The concern about suicide prevention in young people has been increasing over the years despite the efforts dedicated to the subject. University students are a group composed mainly of young people ranging from 18 to 24 years old. According to the World Health Organization, suicide was the fourth cause of death in the 15–24 age group in 2019 [1], and suicide rates (per 100,000 inhabitants) varied between regions and countries. In the Americas region, Latin America showed a lower prevalence of over 100,000 (from 5.7 in Colombia, Peru, and Venezuela to 5–10 in Mexico, Argentina, Brazil, and Chile) compared with Canada (between 10 and 15) and USA (equal or more than 15). For 2019, it was 7.1 over 100,000 in Mexico before the COVID-16 pandemic. Recent data from the National Institute of Statistics, Geography, and Informatic of Mexico [2] shows that Mexico’s global suicide rate (for all ages) went from 5.7 in 2019 to 6.2 in 2020 and peaked in 2021 (the second year of the pandemic) in 6.8 over 100,000, lowering then to 6.3 in 2022. Moreover, it is essential to note that in over ten deaths by suicide in Mexico, eight correspond to men, against two for women. The age group with the highest prevalence was the 20–34 years old group, peaking at 25–29 years (11.6 over 100,000), 11 for 30–35, and 10.6 over 100,000 for the 20–24 group. Inside the country, there are also variations by the federative entity, from 2–4.5 (i.e., Veracruz) to as high as 8.3–11.8 (i.e., Yucatan).

According to Cha et al., suicidal thoughts and behavior include several concepts [3]. In the cognitive domain, based on Beck concept [4], Suicidal Ideation (SI) refers to the consideration or desire to terminate one’s own life, ranging from passive fantasies to being dead to more active thinking about killing oneself, including or not the election of a means to do it (suicidal planning). The behavioral domain involves getting the elements to do it (suicidal preparation) and the action in itself, which could be unsuccessful (suicide attempt) or, when it leads to death, it is referred to as consummated suicide.

Contrary to suicidal behaviors, suicidal ideation is still a broad term that includes suicidal thoughts, ideas, fantasies, and worries about death and has diverse operational definitions in different research studies that could include or not include suicidal planning. For example, Forsyth et al. name “suicidal pre-ideation” the affirmative response to the question of having ever thought of attempting suicide. In contrast, “suicide ideation” requires that people have thought “seriously considering attempting suicide,” defining it as a distinct category from suicidal planning and suicide attempts [5].

Statistics show that around 57 to 80% of suicidal deaths occur in the first attempt. Also, about 50% of attempters or deceased people did not communicate about suicidal thoughts or deny them in previous evaluations [6]. Indeed, assessing previous attempts to evaluate suicidal risk in young people is two steps behind early detection, and asking about suicidal ideation will not always be accurate.

University students are a mental health risk population due to the increased stress linked to multiple transitions. They go through a transition from adolescence to emerging adulthood, sometimes with separation from the family and community of origin to live in a different place, from high school to a more exigent and formal academic environment, facing increased time to solve homework, domestic work, self-care, and working hours to get financial income. University students face these challenges with a different set of personal abilities and resources, as well as unequal vulnerabilities, including the fact that most mental health problems begin around the time of starting university and can be undetected and untreated by the moment of their admission [7]. Currently, university students belong to a particular generation with diverse denominations; one of the most descriptive ones is “digital natives” [8], referring to the fact that they are the first generation to grow up using a variety of digital devices, applications and platforms integrated into their daily lives, that determine profound differences in the way they relate to and communicate with others, think and learn, which warrants inquiring about virtual social networks use and their influence on suicidal ideation.

Suicidal prevention in universities requires accurate detection and risk evaluation, which is complex and challenging because it is multi-determined. Consequently, it could be beneficial to screen not only for suicidal ideation but also the numerous associated risk and protective factors that proved to be influential as predictors of SI [3]: on an environmental level, interpersonal violence such as bullying and cyberbullying had strong support from empirical evidence, as well as psychological factors: predominantly negative affect, low self-esteem and the lack of positive affect. On the social processes level, the lack of connectedness (loneliness) has support as a predictive of suicidal risk, and some research suggests that it could also have an indirect effect as a mediator, i.e., when associated with psychopathology [3].

The bioecological model of human development [9] brings an organized frame to observe the multiple interactions between the subjects (ontosystem) with their particular biological singularity and their developmental environments, from the more proximal ones (microsystem) as family, school, and community, with their intertwined connections (mesosystem), the structural factors (exosystem) that affect microsystems, such as health and social services, and the sociocultural influences (macrosystem) as gender that transversally influence all the other levels. These dimensions have reciprocal interactions, and their changes over time determine changes (co-evolutions) in the other levels (cronosystem).

Looking for a practical way of screening SI altogether with vulnerabilities and protective factors, we selected psychological distress, loneliness, life satisfaction, self-concept (ontosystem), family functioning, perceived community support, bullying victimization, problematic use of virtual social networks (microsystem), and gender (macrosystem) to explore their relations and effect on SI in a group of Mexican university students.

At the Ontosystem level, an essential determinant of SI is psychological suffering or discomfort, characterized as psychological distress. Psychological distress (PD) appears when stress overcomes the coping resources of the subject. The discomfort could vary in intensity, persistence, and repercussion, ranging from subclinical to severe, impacting emotional, cognitive, and behavioral domains of functioning, which could warrant a mental disorder diagnosis [10]. The advantage of looking for PD instead of mental disorders (such as depressive and anxiety disorders, borderline personality disorder, and substance use disorders, among others) is that it allows the detection of psychological suffering before it evolves into a clinical mental disorder. In addition, it is easy to measure in non-clinical settings since the instrument is a self-report scale that does not require specialized knowledge [11].

Among the positive affect variables, life satisfaction has been widely studied as a protective mental health factor, and low life satisfaction is associated with increased suicidal risk [12,13]. Several studies confirmed Self-esteem as a protective factor amongst students’ SI in Mexican universities [14,15]. Self-concept, the cognitive component of self-esteem, differentiates specific domains, such as academic, familial, social, emotional, and physical, and is positively associated with psychological well-being [16], higher life satisfaction, and the microsystemic variables of family functioning and community support [17,18].

At a microsystem level, we included the perception of relational support available, which is especially important when young people face life difficulties and challenges. In Mexico and Latin America, a central component of relational support is the family, followed by community social support [17,18,19]. Opposed to that effect is the perception of loneliness, frequently studied concerning suicidal risk [20,21]. Previous research on Mexican university students showed a protective effect of good family functioning and social support over SI [14,15].

Interpersonal violence is one of the most proven factors increasing the risk of young suicide [3]. Bullying, cyberbullying, internet violence, child maltreatment, and family violence affect mental health, weakening the protective elements (life satisfaction, self-concept, community social support, and family functioning perception) and increasing the risk-related ones (PD, loneliness). School bullying is unprovoked interpersonal violence, persistent over time, and occurs in a power imbalance situation that favors the aggressor [22]. School bullying victimization increases SI in Mexican adolescents [23]. It is associated with suicidal behavior in young [24], and extensive research in this field confirmed the short and long-term impact on young suicidal risk [25].

The problematic use of virtual social networks (VSN) is currently a public health concern due to the increased amount of time dedicated to internet use by young people [26,27]. Several lines of research pointed out its adverse effects on health and mental health in several domains [28]. A central worry is the possibility of compulsive use, fulfilling the criteria for dependence disorder [29], and its links with internet violence and victimization, called cyberbullying [30]. Some evidence showed that cyberbullying could have more impact on suicidal risk than traditional bullying [30,31] and that both forms of bullying could be combined [32]. Some studies have suggested that the effects of internet violence and rejection on SI are mainly indirect, mediated by PD and loneliness [21].

Finally, gender is a macrosystemic variable influencing role norms, rights, expectations, and possibilities for girls, boys, and diverse young. Gender affects suicide vulnerabilities and possibilities of early detection and intervention; therefore, it is crucial to include it for effective suicide prevention. Women and girls worldwide report higher values of SI and PD than men and boys, but boys die because of suicide three to five times more than girls [33]. Diverse gender (LGBTQI+) young people are a subgroup with higher SI and suicidal risk [3], also related to higher violence victimization (structural and interpersonal) and social exclusion.

These factors could interact with each other in complex and bidirectional ways. For example, people with low social self-concept and poor social skills can spend more time interacting on the internet because it seems less challenging for them, at the expense of face-to-face interactions that could improve their social skills and enhance their community support [30,34]. Internet dependence leads to higher screen time, with more involvement in cyberbullying and increased PD [31], which could impact SI. On the other hand, a positive family climate and community support strengthen self-concept and life satisfaction, protecting against compulsive use of VSN and modulating the impact of internet violence/rejection over PD [35].

Considering the previously exposed and the fact that ecological studies in the Mexican university population are scarce, this research aims to explore the correlations between ontosystem, microsystem, and macrosystem factors with SI and test their predictive value on a sample of Mexican university students. We hypothesized that the combination of increased risk factors (PD, loneliness, violence victimization, and inadequate use of VSN as Internet violence/rejection and Internet dependence) with lower protective factors (self-concept, life satisfaction, family functioning, and community support) would allow us to identify the students with a higher risk of SI that requires more thorough evaluation (and early intervention) for suicidal risk. For that purpose, we planned this exploratory quantitative research that includes descriptive basal values in a sample of university students, lineal bidirectional correlations to find the significant and relevant associations between variables, a cluster analysis to classify belonging to groups with different degrees of SI, a first step to performing the discriminant analyses determining the predictive variables, and a multilinear regression model to measure the relative weight of this group of variables in the prediction of SI. Our statistical hypotheses were:

**H1.**  *SI will correlate positively with psychological distress, loneliness, bullying victimization, internet violence/rejection, and internet dependence and negatively with life satisfaction, self-concept, social support, and family functioning.*

**H2.**  *PD, loneliness, bullying-victimization, internet victimization and dependence, life satisfaction, social support, family function, and self-concept will predict SI on the discriminant analyses*.

**H3.**  *Girls will present higher means of PD and SI and lower levels of family functioning than boys, and we will find differences by gender in the predictive value of the variables studied*.

## 2. Materials and Methods

It is a quantitative, observational, non-experimental, cross-sectional study.

### 2.1. Participants

The study was planned for a representative proportional sample of 1446 active students from a public Mexican university (N 6220), stratified by the educative program. The questionnaire’s application was presential, initiated in March 2020, and was interrupted by the mandatory confinement related to the COVID-19 pandemic. Hence, because only students from two programs (Psychology and Physical Culture) participated, it could be considered an incidental sample. Three hundred seventy-eight students agreed to participate (rate of acceptance 99%). Two cases were excluded due to missing data. The final sample was composed of proportional clusters of the career’s initial, middle, and final stages; 67% were women (252), and 33% were men (124) with ages between 18 and 34 years old (M = 20.8).

### 2.2. Procedure

This non-experimental research does not include any procedure that can harm or be prejudicial to participants. It follows the Helsinki bioethics protocol, being reviewed, approved, and authorized by the Departmental College of Psychology Department at the university center.

The researchers selected the groups by education level and randomly chose the participants by numbers for every group. At least one researcher was present during the application, which lasted around one hour, to assist in cases of doubt about the items. After the researchers explained the purpose of the study, warranting anonymity and voluntary participation, the participants signed a consent letter and filled out the self-report questionnaire in their classrooms. Questionnaires and informed consent were foliated for identification. The informed consent included a space for the student code. It was detached from the questionnaire and packed in closed envelopes for contact and follow-up with at-risk students.

### 2.3. Instruments

Congruently with Bronfenbrenner’s ecological model remark on the importance of subjective perception rather than extern evaluation, we used only self-report instruments.

Sociodemographic data included gender, age, residence, educational program, semester, work status, and number of weekly work hours.

The standardized scales included in the questionnaire were:

The F-5 Multidimensional Self-Concept (AF-5) scale by García and Musitu [36] is a questionnaire with a Likert-type response ranging from 1 (never) to 5 (always). It consists of 30 items divided into five dimensions: Academic (items 1, 6, 11, 16, 21, 26, i.e., “My professors think that I am a Good student”), social (items 2, 7, 12, 17, 22, 27, i.e., “I am a friendly person”), emotional (items 3, 13, 18, 23, 28, i.e., “I am easily scared”), family (items 4, 9, 14, 19, 24, 29, i.e., “My family Will help me in any problems”), and physical self-concept (items 5, 10, 15, 20, 25, 30, i.e., “I am an attractive person”). Within this questionnaire, items 3, 4, 12, 13, 14, 18, 22, 23, and 28 require reversing their scores before summation. The reliability in this study, according to Cronbach’s alpha, was 0.84 (Academic), 0.78 (Social), 0.86 (Family), 0.85 (Emotional), and 0.76 (Physical).

Robert’s Suicidal Ideation scale [37] Spanish version adapted by Mariño et al. [14], comprises four items: (a) I could not “move on”; (b) I had thoughts about death, (c) I felt that my family would be better off if I were dead, and (d) I thought about killing myself. It has Likert-type responses indicating the prevalence in the last week, rating from 1 to 4 (1 = 0 days; 2 = 1–2 days; 3 = 3–4 days, and 4 = 5–7 days). It is standardized for the Mexican and Spanish populations. The cutting point indicating suicidal risk was established as equal to or above six by previous research in the Mexican population [38]. Reliability by Cronbach’s alpha was 0.79 in this study.

K10 Psychological distress scale of Kessler and Mroczek [39] Spanish adaptation by Alonso et al. [40], composed of 10 items (i.e., How often have you felt nervous?) exploring psychological discomfort over the last month, with five options, ranging from 1 to 5 (never, rarely, sometimes, often, and always). Total scores are classified into four categories: “no psychological distress” (scores between 10 and 19), “mild psychological distress” (between 20 and 24), “moderate psychological distress” (between 25 and 29), and “extreme psychological distress” (between 30 and 50). Reliability by Cronbach’s alpha was 0.90 in this study.

The Community social support scale by Gracia et al. [41] includes three subscales: Community Implication (i.e., “I am appreciated in my community”), community participation (i.e., “I use to participate in the activities organized in my community”), and informal systems social support (i.e., “In my community there are people that can help me to solve my problems”), with Likers’s type responses from 1 (strongly disagree) to 4 (totally agree). Reliability by Cronbach alpha for this study was 0.64 (Integration), 0.70 (participation), and 0.65 (Informal support).

UCLA Loneliness Scale by Russell et al. [42], adapted to Spanish by Expósito and Moya [43], with Likert-type responses from 1 (never) to 4 (always). It consists of 20 items distributed in two factors: emotional loneliness (items 2, 3, 4, 7, 8, 11, 12, 13, 14, 17, and 18, i.e., “How often do you feel isolated from others?”) and subjective evaluation of the social network (items 1, 5, 6, 9, 10, 15, 16, 19, 20, i.e., “How often do you feel that the people around you understand you?”). Reliability for this study was 0.93 Cronbach alpha.

Virtual social nets use scale ESOC 39 by LISIS group [29], comprising 39 items with Likert-type responses from 1 (Never/Nothing) to 4 (Always/Much). It has two parts: first, seven items providing structural information, and the second with 31 items grouped into four factors: Internet Dependency (i.e.,” If I do not connect to my social network, I feel bad and get in a bad mood”), Internet Violence and Rejection (i.e., ” In my social network I have felt discriminated against for being who I am”), Internet Friendship Strengthening (i.e., “On my social network I keep in touch with friends I have not seen in a while”), and Internet Social Facilitation (i.e., “In my social network I have met new friends”). In this study, Cronbach’s alpha reliability was 0.83 for Internet dependency, 0.65 for Internet Violence and Rejection, 0.75 for Internet friendship and 0.75 for Internet social facilitation.

The School Victimization scale by Cava and Buelga [44] comprehends 22 items with Likert responses from 1 (never) to 4 (always). It is composed of three factors: relational victimization (i.e., “A schoolmate has told others not to be my friends”), physical victimization (i.e., “A schoolmate hit me”), and verbal victimization (i.e., “A schoolmate insulted me”). In this study, only relational (Cronbach’s Alpha = 0.89) and verbal victimization (Cronbach’s Alpha = 0.77) were reliable.

APGAR family function scale by Smilkstein et al. [45], Spanish adaptation by Bellón Saameño et al. [46], contains five positively worded items with Likert responses from 1 (never) to 5 (always), measuring family adaptation, participation, growth, affection, and problem-solving (i.e., “Are you satisfied with the help you receive from your family when you have a problem?”) It represents a single factor: satisfaction with family functioning, with a Cronbach’s alpha reliability of 0.84 in this study.

Life Satisfaction Scale by Diener et al. [47], adapted to Spanish by Atienza et al. [48], uses Likert-type responses from 1 (strongly disagree) to 4 (strongly agree). The scale provides a general index of overall life satisfaction, is composed of five items (i.e., “My life is like I wish it to be in the majority of aspects”), and has a single factor, life satisfaction, with a Cronbach’s alpha reliability of 0.70 for this study.

### 2.4. Statistical Analysis

We treated missing data using the regression imputation method when they accounted for less than 20% of the scale. Above this value, we exclude the scale for the subject and the subject if there were more than two scales excluded. We excluded two subjects for missing data from an initial sample of 378 students, resulting in 376 students.

Descriptive statistics were calculated for subject attributes and expressed as means, ±standard deviation, frequencies, and percentages. The t-Student proof confirmed gender differences in some variables’ means and standard deviations. We calculated the linear association between the continuous variables with Pearson’s product-moment coefficient.

We conducted a Two-Step Cluster Analysis to obtain the natural clusters according to SI, grouping them into No SI, Low SI, Moderate SI, and High SI [49]. To identify the variables that best distinguish between the No SI group and Moderate plus High SI (which we will call the High IS group), we performed a Discriminant analysis with all the variables with significant correlations in the Pearson test. The saturation cut-off for the correlated variables in the structure matrix was equal to or above 0.25 [50].

A multilinear regression analysis was performed to evaluate the associations between the independent variables and SI and corroborate predictive values. The number of cases to warrant liability was calculated following Green estimation for Multiple regression R square medium effect [51] N > or = 50 + 8 K, being K the number of variables processed (11 in this case) and determined in a minimum of 138 participants. Additionally, we processed separated multilinear regression analyses for boys and girls, examining gender differences. The minimal level of significance was *p* < 0.05. For statistical analyses, we used SPSS 25.0 Statistical Software Package for Windows.

## 3. Results

All the participants were actively studying at the university between the first and final semesters.

### 3.1. Descriptive Results

Sociodemographic characteristics are shown in Table 1. A total of 69% of the students were local, and 31% were foreigners. The mean age was 20.9 years. Women predominated over men with almost 67%. A total of 85% of students responded yes to the question, “Are you currently working?” We asked them how many hours they worked weekly, and the mean was 10 h per week, predominating the students that work 1–10 h weekly, with 84%; 12.5% responded that they worked 11 to 20 h weekly, and only 3.4% worked 21 or more h weekly.

Descriptions for each variable are shown in Table 2. Because of the multiplicity of variables, we divided them into ontosystemic and microsystemic variables.

It is noticeable that the higher media for self-concept was family self-concept (24.64), expressing that most university students in this sample consider their families to love and care for them. The lowest media was for Physical Self-Concept, which scored 18.59.

Psychological distress media was 26.36, moderate according to the clinical cutting points, which means that a significant proportion of students present psychological discomfort. Regarding suicide ideation, the media of 5.54 was below the cutting point of six, established as a suicide risk marker [38].

Between the microsystemic variables, the victimization scores were low, especially regarding Physical Victimization (4.9), which also presents the lowest variation (SD = 1) of all variables measured.

The t-Students test calculated differences by gender. Women reported significantly higher means of SI (F = 8.73, *p* = 0.003) and PD (F = 5.81, *p* = 0.016). Men showed significantly higher Physical Self-Concept (F = 27.34, *p* < 0.001), Emotional Self-Concept (F = 30.57, *p* < 0.001), Familial Self-Concept (F = 4.93, *p* = 0.027), and Verbal Victimization (F = 4.35, *p* = 0.038). The remaining variables (Academic Self-Concept, Life Satisfaction, Family Functioning, Loneliness, Community Support, Problematic Use of VSN, and Relational Victimization) did not present significant differences between men and women.

### 3.2. Correlational Analyses

For the first screen of the linear association between the variables and to detect possible multicollinearity (r > 0.80), we calculated Pearson correlations (Table A1). With the exceptions of Internet Friends and Internet Social Facilitation, Community Participation, Verbal Victimization (no significance), and Relational Victimization (significant at 0.05), all the variables correlated with SI at a level of 0.01. The r coefficient was positive for PD (r = 0.67), Loneliness (r = 0.52, Internet Violence/Rejection (r = 0.29), and Internet Dependence (r = 0.19). The r was negative for Family Self-Concept (r = −0.45), Emotional Self-Concept (r = −0.42), Life Satisfaction (r = −0.38), Family Functioning (r = −0.35), Physical Self-Concept (r = −0.34), Social Self-Concept (r = −0.26), Community Informal Support (r = −0.26), and Academic Self-Concept (r = −0.22).

All the variables correlated significantly with psychological distress at the 0.01 level, except Internet Friends (no significance) and Community Participation (significance at 0.05). PD correlated positively with Loneliness (r = 0.67), and Internet Violence and Rejection (r = 0.35) and negatively with Emotional Self-Concept (r = −0.61), Family Self-Concept (r = −0.51), Life Satisfaction (r = −0.48), and Family Functioning (r = −0.45).

Loneliness correlated significantly at 0.01 level with all the variables, except Internet Dependence, Internet Friends and Internet Social Facilitator (not significant), and Verbal Victimization (significant at 0.05 level). The most relevant correlations of Loneliness were with Social Self-Concept (r = −0.60), Family Self-concept (r = −0.55), Life Satisfaction (r = −0.54), Family Functioning (r = −0.54), and Community Informal Support (r = −0.53).

Life satisfaction correlated significantly at 0.01 level with all the variables except Internet friends, Internet Social Facilitation, Verbal Victimization (no significance), and Relational Victimization (significant at 0.05). The most relevant correlations were the negative ones already mentioned with SI and PD, with the highest r value with Loneliness (r = −0.54). Positive, more relevant correlations were with Family Self-Concept (r = 0.49), Family Functioning (r = 0.33), and Informal Social Support (r = 0.33).

Internet dependence correlated significantly at 0.01 level with all the variables, except with Academic, Social, and Physical Self-Concept, Family Functioning, the three subscales of Community Social Support (not significant), and Life Satisfaction (significant at 0.05 level). Its correlations were small, with the highest positive correlations with PD (r = 0.24) and with the other subscales of ESOC, Internet Friends (r = 0.40), Internet Social Facilitation (r = 0.49), and Internet Violence and Rejection (r = 0.50).

Internet Violence Rejection correlated significantly at 0.01 level with all the variables except Family functioning. The three subscales of Community Participation (no significance), Internet Friends, Internet Social Facilitation, and Community integration (significant at 0.05 level); the highest was the positive correlations with Verbal Victimization (r = 0.31), and the negative correlations with Emotional (r = −0.34), and Family Self-Concept (r = 0.30).

Internet friends correlated significantly at 0.01 level only with Social Self-Concept (r = 0.20), Internet Dependence (r = 0.40), and Internet Social Facilitation (r = 0.49). There was also a small positive correlation with Community Informal Support (r = 0.18).

Internet Social Facilitation correlated significantly at 0.01 level only with Emotional Self-Concept (r = −0.16), Internet Dependence (r = 0.49), Internet Violence Rejection (r = 0.27), and Internet Friends (r = 0.49). There was also an intriguing small positive correlation with PD (r = 0.14).

Community integration correlated significantly at 0.01 level with all the variables except Internet dependence, Internet Social Facilitation, Relational Victimization (no significance), Internet Violence and Rejection, and Internet Friends (significant at 0.05 level). Its most relevant correlations were negative with Loneliness (r = −0.39) and positive with Life Satisfaction (r = 0.40), Social Self-Concept (r = 0.36), and Physical Self-Concept (r = 0.30).

Community participation correlated significantly at 0.01 with Loneliness (r = −0.39) with all the self-concept variables, Life Satisfaction, Family Functioning, and Verbal Victimization. Correlations with Suicidal Ideation, Internet Dependence, Internet Social Facilitation, Internet Violence and Rejection, Internet Friends, and Relational Victimization were not significant, and the correlation with PD was significant at 0.05 level. Its most relevant correlation was positive, with Physical (r = 0.43), Academic (r = 0.32), and Social Self-Concept (r = 0.32).

Community informal support correlated significantly at 0.01 level with all variables except Internet Dependence, Verbal Victimization (not significant), and Internet Social Facilitation (significant at 0.05 level). Its most relevant correlation was positive, with Academic (r = 0.32) and Social Self-Concept (r = 0.32), Life Satisfaction (r = 0.33), and negative with Loneliness (r = −0.53) and PD (r = −0.33).

Verbal Victimization correlated significantly at 0.01 level only with PD, Loneliness, Internet dependence, and Internet violence/rejection, its only relevant correlation (r = 0.31), and Loneliness, Internet Social Facilitation, and Family functioning (significant at 0.05 level).

Finally, Relational Victimization correlated significantly at 0.01 level with PD, Loneliness, Family Self-Concept, Internet Dependence, Internet Violence/Rejection, Family Functioning, Community Informal Support, and Verbal Victimization. Correlations with SI and Physical Self-concept were significant at 0.05 level. Its most relevant correlation was with Internet violence/rejection (r = 0.26), aside from the positive correlation with the Verbal Victimization subscale (r = 0.73).

### 3.3. Cluster Analyses

In order to find the maximum intra-group similarities and maximum differences between groups, we performed a two-step cluster analysis. The test identified four groups of participants with different levels of SI: No SI, Low SI, Moderate SI, and High SI. As Table 3 shows, participants in the No SI group (156 students) represent 41.3% of the sample, with a median of 4. The value corresponds to adding the response” zero-day in last week” to the four items of the Roberts Suicidal Ideation scale. The group with Low SI was 38.1%, corresponding to 144 students, with a median of 5. 27, below the cutting point for suicidal risk of six. The cluster Moderate SI accounts for 15.9% (60 students) with a median of 7.97, above the cutting point for suicidal risk. Finally, the High SI cluster accounted for 4.8 (18 students) with a median of 12.83. The total number of students with some Suicidal Ideation risk, measured with the Roberts scale, was 20.7% for this study, corresponding to 78 students.

### 3.4. Discriminant Analyses

To find the linear combination of the variables that best differentiate the scores in the independent variable (Suicidal ideation), we performed a Discriminant analysis including the No Suicidal Ideation cluster (156 students) and the Moderate plus High SI clusters (78 students), that had media above 6 (suicidal risk). We will call it the High SI cluster. The two groups had statistically significant differences in SI (Table 4).

The students without SI scored significantly higher in Self-Concept (Academic, Social, Emotional, Familial, and Physical), Life Satisfaction, Family Functioning, Community Integration, Community Participation, and Informal Social Support. Conversely, the students with High SI presented higher Psychological Distress, Loneliness, Violence and Rejection In VSN, dependency on VSN, and Relational Victimization at school.

Table A2 shows each variable’s mean, standard deviation, and t F values according to the cluster. Differences between No SI and High SI clusters were statistically significant for all variables except for Virtual Social Net friends, Social Net Social Facilitation, and Verbal Victimization.

The M de Box test was significant (F (5,567,201.507) = 99.513; *p* < 0.001), discarding the null hypothesis that the matrixes of population covariance were equal. The Wilks lambda coefficient was significant (X2(11) = 200.587; *p* < 0.000), which rejected the null hypothesis of equality between the clusters in the means of the discriminant variables. The canonic correlation coefficient was η2 = 0.77, confirming the model’s validity to discriminate between the two clusters. Table 5 shows the coordinates of the centroid projection of each group over the discriminant function. The coefficients indicate the number of standard deviations in that the means of each group deviate from the central mean for the sample. Because the distribution is normalized, the mean is 1, and the sigma is 0. In the resulting functions, the No SI group deviates from the center in the opposite direction of the High SI group.

The matrix structure with the variables, ordered by degree of canonic correlation (saturation) with the discriminant function, is shown in Table 6. The cutoff point is highlighted. Although the variables Community Integration, Relational Victimization, Internet Dependence, Community Participation, and Verbal Victimization are present, their values below the established cutoff point of 0.25 indicate they are less relevant as predictors.

The variables predicting High SI were psychological distress, loneliness, internet violence/rejection, and dependency. The variables that predicted the absence of SI were Family Self-Concept, Life Satisfaction, Emotional Self-Concept, Family Functioning, Physical Self-Concept, Community Informal Support, Social Self-Concept, and Academic Self-Concept.

In order to test the prediction accuracy of the discriminant variables, we performed a Classification Test. The discriminant model correctly classified the cases belonging to the No SI group in 92.9% of the cases and those belonging to the High SI group in 78.2%. In total, 88% of the cases grouped were classified correctly.

### 3.5. Multilinear Regression

The Multilinear Regression Model (Table 7), including the discriminant variables, had an excellent adjusted R-square (0.457) and identified Psychological Distress (B = 0.14, *p* = 0.000, 95% CI: 0.11, 0.18) as predictive of higher Suicidal Ideation, and Familial Self-Concept (B = −0.09, *p* = 0.013, 95% CI −0.15, −0.02), as predictive of lower SI. The other variables did not reach statistical significance.

The linear regression model with men as a subgroup (n = 122) and SI as a dependent variable (Table A2) had an excellent adjusted R-square (0.337). The results showed that levels of SI were associated with the levels of PD (B = 0.07, *p* = 0.009, 95% CI: 0.02, 0.12) and Physical Self-Concept (B = −0.08, *p* = 0.050, 95% CI: −0.15, −0.00). The model for women (n = 253) presented an excellent adjusted R-square (0.494). The results showed that levels of SI were associated with the levels of PD (B = 0.17, *p* < 0.001, 95% CI: 0.13, 0.21) and Family Self-Concept (B = −0.08, *p* = 0.050, 95% CI: −0.16, −0.00), as it showed in Table A3.

## 4. Discussion

This study aimed to explore suicidal ideation and the associated factors in individual (ontosystem), family, and community, including virtual social media socialization (Microsystem) and gender (macrosystem) in a sample of Mexican university students, to find a way to broadly screen a combination of early indicators of suicidal risk, that can predict suicidal ideation, and guide contextualized prevention and early risk intervention. For this purpose, we used easy self-report standardized scales that enable us to perform periodic evaluations and longitudinal studies in the university population.

### 4.1. Result’s Discussion

Descriptive statistic shows that the media for SI in the sample was 5.54, below the cutting point of six established in Mexican adolescents [38] for the scale used in this study. The prevalence of suicidal risk, measuring only last week’s Suicidal thoughts, not including suicidal planning or attempts, was 22%, coincidentally with the most prominent research in a Mexican university conducted in 2019 by Benjet et al., with broad SI measure [52] and also with Mortier et al. meta-analyses of pooled lifetime prevalence of suicidal thoughts in college students, published in 2018 [53]. Our 22% prevalence included Moderate and High suicidal ideation clusters. Only 4.8% accounted for the High SI group, excluding suicidal planning and attempts.

The sociodemographic data inform that 85% of participants work; 84% work between 1–10 h weekly, corresponding partial time and informal employs, or self-employment (i.e., micro-sales of food at the university campus). Only 3.4% worked full-time (21 or more hours weekly). Even when it is common for students in Mexican public universities to have a part-time job to complement the economic family support, the ones that depend entirely on their incomes are the fewer. Unfortunately, we do not have specific university student data to contrast these values. However, the last reports of a Mexican official source from 2020 [54] indicate that approximately 57% of young between 15 and 29 years old are economically active, with the unemployment rate for this age group of 6.2. Previous research on sociodemographic variables related to increased suicidal risk in Mexican university students found that while the income insufficient to cover their needs increased suicidal risk, working has a protective effect over suicidal risk [15]. A recent study [55] pointed out that a rise in economic uncertainty elevates suicidal risk in high-income countries but not in middle or low-income countries, such as in Mexico. Our students’ working status and weekly hours should be included in estimating income sufficiency in subsequent research to evaluate the influence of these macrosystem variables on the microsystem and ontosystem. This study’s correlation of working hours with SI did not reach statistical significance.

The most relevant positive correlations with SI were at the ontosystem level, with the individual variables PD and Loneliness confirming previous findings in other countries [56,57]. Both, in turn, correlated positively with microsystemic variables such as Internet Violence/Rejection, Internet Dependency, and Relational Bullying Victimization. Negative most relevant correlations with SI followed the same distribution, being the highest with the ontosystemic variables Self-Concept (especially Family Self-Concept) and Life Satisfaction. Microsystemic variables were in the same direction, especially Family Functioning and Community Informal Social Support. These associations confirmed previous results in another context [58,59] and local research with early adolescents [23]. Following the line of correlations, we can confirm the inverse relation of microsystem over the factors related to higher SI (PD and loneliness) being Family Functioning r coefficient higher than Community Supports. These results highlight the protective effect over SI of a good family functioning, directly and indirectly through Family Self-Concept. Worth of notice is the positive and relevant correlation between Family Function and Life Satisfaction, as well as their negative correlation with both ontosystemic risk factors, Psychological Distress, and Loneliness. Community Support, in turn, correlated negatively with SI, particularly Informal system support, which shows relevant correlations with Loneliness and PD and positively with Academic, Social, and Physical Self-Concept.

In general, the factors of the virtual social nets use scale presented small correlations with the other variables, except with same scale variables, particularly between Internet Dependence and Internet Violence/Rejection. Internet Dependence most correlated positively with PD and Emotional Self-Concept, and with Verbal and Relational Victimization, always with r coefficients around 0.2 or lower. The correlation of SI and Internet Friends and Social Facilitation did not reach statistical significance. Even when the r coefficient was small (r = 0.12, *p* < 0.01), Internet Social Facilitation positively correlated with Psychological Distress. It also shows a positive correlation with Informal Community Support (r = 0.11, *p* < 0.01) and a negative correlation with Emotional Self-Concept (r = −0.14, *p* < 0.001). This result could be explained by previous findings suggesting that young people with social difficulties tend to rely more on internet interactions, enhancing the likelihood of increased Psychological Distress and Loneliness by higher exposure to violence and rejection in virtual social nets, as documented in previous research [60]. Internet Violence and Rejection was the subscale that shows the more relevant positive r values in relation with SI, PD and loneliness, and negatively with Emotional and Family Self-Concept, as well as with the three variables of community support. Its positive relation with Verbal and Relational Victimization was also noticeable. Taking into account their belonging to the digital native generation, we actually expected to find more robust evidence about the effect of virtual social media on the SI and PD, but these results suggest that, at least in our population, the main influences on university students remain in the self-concept, family and community domain.

We expected that PD, Loneliness, Bullying Victimization, Internet Victimization and Dependence, Life Satisfaction, Community Social Support, Family Function, and Self-Concept would discriminate the presence of high SI or its absence (H2).

Discriminant analyses proved that PD, Loneliness, and Internet Violence/Rejection (but not dependence) predicted high suicidal ideation. Conversely, Life Satisfaction, Multidimensional Self-Concept, Family Functioning, Community Informal Support (but no community integration and participation) predicted No SI.

The most robust predictive variable was psychological distress. Loneliness was not predictive in the multilinear regression. This result supports previous findings showing that loneliness per se does not increase suicidal risk [3], but it does when is associated with psychopathology or bullying [61]. In consequence, we propose to consider increased suicidal risk in young people who present high values of PD associated with loneliness. Internet Violence/rejection and SI correlated higher in this group than traditional bullying, suggesting that face-to-face violence could be less frequent or influential for university students than younger adolescents. More important than age, the exposition to cyberbullying seems related to the time spent online and the types of technologies used [34].

All five self-concept dimensions predicted no suicidal ideation, congruent with previous findings in Mexican university students [14,57]. The failure of these protective factors can turn them into a risk factor that increases SI [61] as the perception of low family functioning, lack of support and help, communication, and time to spend together.

This result is congruent with a previous report [62] showing that, even when School/career is the most prevalent source of stress in adolescents, its impact on PD and SI is lower than interpersonal factors such as conflicts with parents, peers, and family circumstances. At a microsystem level, the perception of good Family Functioning and Informal Social Support were predictive of belonging to the No SI group. Inadequate coping with PD includes the increased use of VSN and non-suicidal self-injuries. Hence, encouraging healthier strategies to cope with PD, such as spirituality, physical activity, and exercise, will increase suicidal resilience [63].

Our last Hypothesis (H3), that girls will present higher means of PD and SI and lower levels of family functioning than boys, and we will find differences by gender in the predictive value of the variables included, was confirmed in this study. Girls showed significantly higher means in PD and Suicidal Ideation and lower medians of Family, Physical, and Emotional Self-Concept. There were no differences by gender in Social and Academic Self-Concept. In the multilinear regressions, only Psychological Distress and Family Self-Concept predicted SI. We have to remember that predictive power would widely vary according to the variables introduced; this means that of all the eleven variables processed, these two are the ones that show higher B coefficients and significance in this particular set. Aside from PD, Physical Self-Concept was a negative predictor of SI for boys, and Familial Self-Concept for girls.

Previous findings in Mexican early adolescents find that girls perceive more family problems and evaluate their Family Functioning and Self-Concept lower than boys [64]. Family Self-Concept, reflecting a harmonious Family Functioning, would protect girls from SI, while a good Physical Self-Concept would be more important for boys. The last is a striking result, considering that this subscale comprehends the perception of physical attractiveness, strength, and ability. In that sense, the sample’s composition (only Psychology and Physical culture) could be biased since men Psychology students were only 28%, while Physical culture students were 67% at the date of data collection. Physical culture students could have higher Physical Self-Concept than students from other programs [65]. While some evidence pointed out the protective effect of sports practice on suicidal risk [66], which could be mediated by self-esteem and social support, there is not much research about the subject. In our study, Physical Self-Concept has the highest correlations with Community Participation (r = 0.43, *p* < 0.01). Looking at its relevant negative correlation with Loneliness (r = −0.44, *p* < 0.01) it would be worth of further exploration, given the fact that the university offer a variety of physical recreative activities, as well as sport practices, that can be capitalized to strengthen these interrelated protective factors at ontosystem (physical and social self-Concept) and microsystem level (facilitating social inclusion) in the students at risk.

We consider these gender differences linked to traditional patriarchal gender rules that stress obedience and responsibility in girls. Unequal exigencies (i.e., responsibility in domestic work and caring, control over social life, and permissions) lead to more conflicts and dissatisfaction for girls, especially with their mothers. On the other side, traditional gender stereotypes could prevent men from acknowledging their vulnerabilities and emotions, accepting their needs for help, and seeking psychological and psychiatric attention [67]. Gender differences should be included when evaluating suicidal risk and addressed through gender-sensitive family counseling to strengthen prevention and early interventions.

### 4.2. Study Limitations

The limitation of this study relates to the accidental small sample size and the cross-sectional design, which do not allow for causality to be proved. Hence, these results should be taken cautiously and contrasted with broader samples adequate to test theoretical models with structural path analysis and in longitudinal studies. Furthermore, there is a higher prevalence of girls (67%) and initial semester students (40%), proportional to the career composition and course that should be considered for interpreting the results. In the data included in the questionnaire, we did not search for specific psychiatric diagnoses, including substance use disorders.

Because of the importance and frequency of substance use in the university student population and its relevance regarding suicidal risk [60], we acknowledged the need to include at least a broad estimation item about alcohol and other substance use in subsequent research. Contrasting the present results in clinical samples and combining them with qualitative methods will allow us a better and deeper understanding of the dynamics of the interconnected variables and document the results and effectiveness of documented interventions in longitudinal follow-up. We looked for a combination of variables (increased risk factors with low protective factors) that could identify suicidal ideation risk to screen for suicidal ideation periodically. We considered that exploring the identified influential factors among university students in our particular context should be a previous step to designing more accurate research with adequate samples, where mediation/moderation models and path analyses could be useful for theoretical purposes.

### 4.3. Potential Implications for Practice

Taking care of university students’ mental health, including managing the suicidal risk, is still an understudied field characterized by heterogeneity and a lack of consensus [68,69]. The responsibilities and limits of university institutions concerning students’ mental health are still undefined [70]. The multiplicity of issues to consider is overwhelming, and providing evaluation, advice, and support for a growing population seems unreachable. The bioecological model is useful in examining the level of required interventions that could concurrently impact desirable changes and help to find plausible solutions.

While structural and sociocultural changes are less immediate and accessible for most of us, changes in the relations between the onto-system (students) and microsystems (family and community, including universities and virtual social networks) are immediately within reach. It is beyond the scope of this paper to refer in detail to the design of progressive action strategies and plans aiming at a practice informed by evidence that requires a continuous dialogue between basic and applied research. Nevertheless, we could initiate micro-actions at these levels with the preliminary evidence collected from this study.

In this scenario, an ecological approach has the advantage of being adaptable to the particularities of the contexts, individuals, and their resources. Because of its multidimensional nature, it is adequate to evaluate and inform at different levels, from particular individual interventions as well as strategies pointing at the microsystems involved (family, classrooms, university community) to structural and sociocultural aspects involved, and it helps to visualize the dimension in which a particular action should be taken.

Despite its limitations, these research results allow us to stress the relations between SI, PD, and Loneliness, and their connections with the individual (Self-Concept, Life Satisfaction) and relational factors (Family, Community Informal Social Support, and VSN Use) that can be protective or prejudicial. The centrality of caring in prevention and early intervention strategies will simultaneously strengthen protective factors and lower the risks. Individual protective factors will improve through warm and supportive relational environments that are inclusive and nonviolent, with adults attentive to caring for our youngsters. Enhancing good practices of co-responsible caring will be beneficial for mental health promotion and prevention.

Such strategies should arise from those who know the day-to-day situations and dynamics and their particularities to be effective and sustainable. Community involvement is a critical element in the current mental health crisis; therefore, assuming responsibly the need to self-care and to care for others could start micro-actions that extend naturally, becoming sustainable good practices. Finally, periodic evaluations with easy self-report scales in this research would facilitate detecting students who need further evaluation and follow-up [71].

## 5. Conclusions

Suicidal ideation in Mexican university students is strongly related to the degree of PD, Loneliness and Internet Violence and Rejection, and the presence of ontosystemic (Self-Concept, Life Satisfaction) and microsystemic resources (family, communitarian, and virtual social networks), which can also increase or diminish PD and loneliness. The design, launch, and follow-up of structural strategies and programs to address mental health problems, violence, and suicide among university students are indispensable. However, it is also valuable to consider the micro-actions at a relational level that each person can start from their diverse positions and roles in universities. Moving to the encounter with others or being available to initiate and sustain simple but specific actions that add to the young’s informal support (community nets) can start positive changes and co-evolutions. Micro-actions require and depart from accurate local-context knowledge, with their resources and opportunities for down-to-top development, which can warrant sustainability. It seems of particular importance, facing the post-COVID crisis in mental health (which has highlighted the long-lasting debt of structural resources for mental health worldwide), to remember that human beings are capable of agency. Taking even the smallest action to reconnect and rebuild our relational support (strengthening the mesosystem) will enhance communitarian and personal resources and resilience, improving the balance between the increasing stress of our times and the resources to cope with it.

At the specific university-based prevention level, simple but accurate self-report instruments such as those we used in this study can help identify the students at risk earlier, directing them to an accurate evaluation of both psychopathology and suicidal risk and consequent support and specialized intervention. Finally, it can be stressed enough the importance of nutritious and warm relational climates at family, school, and community since empirical evidence shows that interpersonal violence and social isolation are the most influential psychosocial factors related to young suicide. In that sense, the dynamic and impact of the use of virtual social networks require more attention and study because of their wide generalization and increased time of use among the digital native generation.

## Figures and Tables

**Table 1:** Sociodemographic description of the sample. The percentages correspond to the predominant group over the total sample of 378 students (females, local, working, first semester).

Variables	N = 378 (%)
Age (years)	20.9 ± 0.9
Gender (female)	252 (66.9%)
Origin status (local)	261 (69.0%)
Working status (working)	321 (84.9%)
Semester (first)	152 (40.6%)

**Table 2:** Descriptive statistic per variable (N 378): Minimum (Min) and Maximum (Max) scores, Media, and Standard Deviation. Ontosystemic variables included the five factors of the Self-concept F5 Scale, Life Satisfaction, Loneliness, Psychological Distress, and Suicidal Ideation. Microsystemic variables included three factors of the community social support scale, Family Function (Family Apgar scale), four ESOC scale factors, and three school victimization scale factors.

	Min	Max	M	SD
Ontosystemic variables				
Academic Self-Concept	10	30	20.32	3.62
Social Self-Concept	8	30	21.82	4.16
Emotional Self-Concept	6	30	19.32	4.32
Family Self-Concept	10	30	24.64	4.54
Physical Self-Concept	7	30	18.59	4.59
Life Satisfaction	6	20	14.24	2.69
Loneliness	21	73	44.83	10.54
Psychological Distress	11	50	26.36	8.13
Suicidal Ideation	4	16	5.54	2.20
Microsystemic Variables				
Community Integration	9	20	15.04	2.19
Community Participation	6	24	15.42	3.25
Informal Social Support	15	34	26.27	3.44
Family Function	7	25	18.24	4.24
Internet Dependence	13	45	21.59	5.14
Internet Friendship	8	28	20.43	3.82
Internet Social Facilitation	5	19	10.14	2.81
Internet Violence Rejection	7	20	9.42	2.40
Relational Victimization	1	33	13.20	4.14
Physical Victimization	4	11	4.90	1.03
Verbal Victimization	6	19	7.78	2.25

**Table 3:** Clusters of Suicidal Ideation (N 376) obtained by two-step cluster analysis.

	N (%)	M (SD)
No SI	156 (41.3)	4
Low SI	144 (38.1)	5.27
Moderate SI	60 (15.9)	7.97
High SI	18 (4.8)	12.83

**Table 4:** Descriptive Statistics and Difference of Means between Discriminant sub- sample Clusters * (N 234).

	N (%)	M (SD)	F	*p*
No Suicidal Ideation	156 (66.7)	4.00 (0.00)	729.562	0.000
Moderate + High Suicidal Ideation	78 (33.3)	9.09 (2.36)		

* Obtained by Two-Step cluster analysis.

**Table 5:** Coordinates of Centroids projections: (discriminant canonic no standardized functions evaluated by group’s medians).

2 Clusters IS	Function
	1
No SI	−0.840
High IS	1.681

**Table 6:** Main Results of the Discriminant Analysis (structural coefficients): Correlations inside combined groups between discriminant variables and discriminant standardized canonic functions. Variables are ordered by the correlation’s absolute size inside the function.

Psychological Distress	0.901
Loneliness	0.634
Family Self-Concept	−0.513
Life Satisfaction	−0.472
Emotional Self-Concept	−0.451
Family Functioning	−0.391
Physical Self-Concept	−0.383
Internet Violence/Rejection	0.338
Community Informal Support	−0.302
Social Self-Concept	−0.301
Academic Self-Concept	−0.260
Community Integration	−0.218
Relational Victimization	0.172
Internet Dependence	0.167
Community Participation	−0.130
Verbal Victimization	0.092

**Table 7:** Multilinear Regression Model Coefficients (Dependent variable: SI).

95% CI				
	B	*p*	Lower	Upper
(Constant)	2.731	0.135	−0.86	6.32
SS	−0.01	0.784	−0.06	0.05
ES	0.00	0.998	−0.05	0.05
**FS**	**−0.09**	**0.006**	**−0.15**	**−0.02**
PS	−0.04	0.092	−0.08	0.01
LS	−0.02	0.630	−0.10	0.06
Lon	0.02	0.283	−0.01	0.05
CIS	−0.00	0.898	−0.06	0.05
AS	0.03	0.342	−0.03	0.08
IVR	0.02	0.614	−0.06	0.10
**PD**	**0.14**	**0.000**	**0.11**	**0.18**
FF	0.05	0.113	−0.01	0.12

The variables with significant B coefficients are highlighted in bold. SS, Social Self-Concept; ES, Emotional Self-Concept; FS, Family Self-Concept; PS, Physical Self-Concept; LS, Life Satisfaction; Lon, Loneliness; CIS, Community Informal Support; ID, Internet Dependence; AS, Academic Self-Concept; IVR, Internet Violence and Rejection; PD, Psychological |Distress; FF, Family Functioning.

## Data Availability

Data supporting reported results can be consulted by request to correspondent author darsilvananunez@gmail.com.

## Appendix A

**Table A1:** Pearson’s R coefficients and mean differences by gender.

	**SI**	**PD**	**Lon**	**AS**	**SS**	**ES**	**FS**	**PS**	**LS**	**ID**	**IVR**	**IFr**	**ISf**	**FF**	**CIn**	**CPa**	**CIS**	**VV**	**RV**
SI	1																		
PD	0.67 **	1																	
Lon	0.52 **	0.67 **	1																
AS	−0.22 **	−0.29 **	−0.41 **	1															
SS	−0.26 **	−0.29 **	−0.60 **	0.39 **	1														
ES	−0.42 **	−0.61 **	−0.48 **	0.27 **	0.33 **	1													
FS	−0.45 **	−0.51 **	−0.55 **	0.31 **	0.31 **	0.30 **	1												
PS	−0.34 **	−0.38 **	−0.44 **	0.40 **	0.35 **	0.37 **	0.35 **	1											
LS	−0.38 **	−0.47 **	−0.54 **	0.28 **	0.22 **	0.25 **	0.49 **	0.32 **	1										
ID	0.19 **	0.24 **	0.13 *	−0.07	−0.02	−0.22 **	−0.14 **	−0.07	−0.14 *	1									
IVR	0.29 **	0.37 **	0.36 **	−0.12 *	−0.21 **	−0.34 **	−0.30 **	−0.21 **	−0.28 **	0.50 **	1								
IFr	−0.07	0.09	−0.13	0.10	0.20 **	−0.11 *	0.11 *	0.10	0.06	0.40 **	0.12 *	1							
ISf	0.05	0.16 **	0.03	−0.02	0.13 *	−0.14 **	−0.05	0.04	−0.03	0.49 **	0.27 *	0.49 **	1						
FF	−0.35 **	−0.45 **	−0.54 **	0.30 **	0.31 **	0.27 **	0.77 **	0.38 **	0.44 **	−0.09	−0.27 **	0.09	0.00	1					
CIn	−0.19 **	−0.25 **	−0.39 **	0.40 **	0.36 **	0.21 **	0.20 **	0.30 **	0.28 **	−0.04	−0.13 *	0.12 *	0.08	0.19 **	1				
CPa	−0.09	−0.13 *	−0.23 **	0.32 **	0.32 **	0.18 **	0.15 **	0.43 **	0.15 **	−0.02	−0.10	0.09	0.11	0.20 **	0.40 **	1			
CIS	−0.26 **	−0.33 **	−0.53 **	0.31 **	0.37 **	0.26 **	0.29 **	0.19 **	0.33 **	−0.03	−0.14 **	0.18 **	0.13 *	0.29 **	0.51 **	0.28 **	1		
VV	0.09	0.14 **	0.12 *	−0.07	0.03	−0.03	−0.14 *	−0.07	−0.17 **	0.18 **	0.31 **	0.08	0.12 *	−0.11 *	−0.14 **	0.02	−0.08	1	
RV	0.12 *	0.20 **	0.22 **	−0.06	−0.06	−0.06	−0.21 **	−0.13 *	−0.17 **	0.14 **	0.26 **	0.08	0.07	−0.17 **	−0.09	0.01	−0.14 **	0.73**	1
	**SI**	**PD**	**Lon**	**AS**	**SS**	**ES**	**FS**	**PS**	**LS**	**ID**	**IVR**	**IFr**	**ISf**	**FF**	**CIn**	**CPa**	**CIS**	**VV**	**RV**
Men M (SD)	5.07 (1.64)	24.95 (7.34)	44.59 (10.1)	19.96 (3.36)	22.34 (4.07)	21.25 (4.38)	25.36 (3.85)	20.29 (4.39)	14.02 (2.82)	21.94 (5.66)	9.47 (2.48)	20.23 (3.58)	10.51 (2.90)	18.78 (4.02)	15.13 (2.17)	15.57 (3.22)	26.28 (3.44)	8.13 (2.47)	13.35 (4.44)
Women M (SD)	5.78 (2.40)	27.10 (8.44)	44.98 (10.8)	20.51 (3.74)	21.53 (4.18)	18.38 (4.85)	24.26 (4.81)	17.74 (4.46)	14.35 (2.64)	21.42 (4.89)	9.41 (2.37)	20.55 (3.96)	9.96 (2.77)	17.95 (4.32)	15.00 (2.21)	15.34 (3.28)	26.23 (3.43)	7.62 (2.13)	13.13 (4.01)
F	8.73 **	5.81 *	0.11	1.90	3.20	30.57 ***	4.93 *	27.34 ***	1.23	0.84	0.05	0.54	3.14	3.17	0.30	0.43	0.02	4.35 *	0.22

Statistical significance *** = *p* ≤ 0.001; ** = *p* ≤ 0.01; * = *p* ≤ 0.05. M: media; SD Standard deviation. SI, Suicidal Ideation; AS, Academic Self-Concept; SS, Social Self-Concept; ES, Emotional Self-Concept; FS, Family Self-Concept; PS, Physical Self-Concept; LS, Life Satisfaction; Lon, Loneliness; CIn, Community Integration; CPa, Community Participation; CIS, Community Informal Support; ID, Internet’s Dependence; IVR, Internet violence and Rejection; IFr, Internet Friends; ISf; Internet Social facilitation; PD, Psychological distress; FF, Family Functioning; VV, Verbal Victimization; RV, Relational Victimization.

**Table A2:** Differences between No SI and High SI, including variable’s mean, standard deviation, and t F values according to the cluster.

	No SI	High SI		
	M (SD)	M (SD)	F	*p*
AS	21.43 (3.35)	19.09 (3.73)	23.47	0.000
SS	23.39 (3.54)	20.44 (4.26)	31.46	0.000
ES	21.35 (4.20)	16.17 (4.90)	70.72	0.000
FS	26.51 (3.52)	21.09 (5.02)	91.55	0.000
PS	20.65 (4.33)	16.33 (4.42)	51.04	0.000
LS	15.46 (2.43)	12.44 (2.54)	77.57	0.000
Lon	39.06 (8.73)	53.97 (9.79)	139.87	0.000
Cint	15.52 (2.18)	14.29 (2.17)	16.52	0.000
Cpa	16.06 (3.26)	14.97 (3.23)	5.86	0.016
CIS	27.28 (3.07)	24.64 (3.91)	31.75	0.000
ID	20.78 (4.93)	23.06 (6.04)	9.74	0.002
IVR	8.66 (1.84)	10.58 (2.77)	39.63	0.000
Ifr	20.65 (3.82)	20.09 (4.26)	1.04	0.309
Isf	10.04 (2.74)	10.37 (3.12)	0.70	0.404
PD	21.23 (5.51)	35.40 (7.10)	281.96	0.000
FF	19.79 (3.78)	15.79 (4.29)	53.11	0.000
VV	7.55 (2.06)	8.08 (2.51)	2.93	0.088
RV	12.45 (3.59)	14.29 (5.09)	10.29	0.002

Note: SI, Suicidal Ideation; AS, Academic Self-Concept; SS, Social Self-Concept; ES, Emotional Self-Concept; FS, Family Self-Concept; PS, Physical Self-Concept; LS, Life Satisfaction; Lon, Loneliness; CInt, Community Integration; CPa, Community Participation; CIS, Community Informal Support; ID, Internet’s Dependence; IVR, Internet violence and Rejection; IFr, Internet Friends; ISf; Internet Social facilitation; PD, Psychological distress; FF, Family Functioning; VV, Verbal Victimization; RV, Relational Victimization.

**Table A3:** Multilinear regression model for men.

B and *p* Coefficients (Dependent Variable: Men SI).				
95% CI				
	B	*p*	Lower	Upper
(Constant)	4.00	0.192	−2.03	10.04
SS	−0.03	0.508	−0.11	0.05
ES	0.00	0.967	−0.08	0.08
FS	−0.06	0.275	−0.16	0.05
PS	−0.08	0.05	−0.15	0.00
LS	0.01	0.808	−0.10	0.13
Lo	0.03	0.249	−0.02	0.08
CIS	0.00	0.973	−0.09	0.09
AS	0.02	0.663	−0.07	0.10
IVR	−0.02	0.794	−0.13	0.10
PD	0.07	0.009	0.02	0.12
FF	0.06	0.193	−0.03	0.16

Note: SS, Social Self-Concept; ES, Emotional Self-Concept; FS, Family Self-Concept; PS, Physical Self-Concept; AS, Academic Self-Concept; LS, Life Satisfaction; Lo, Loneliness; CIS, Community Informal Support; IVR, Internet violence and Rejection; PD, Psychological distress; FF, Family Functioning.

**Table A4:** Multilinear regression model for women.

B and *p* Coefficients (Dependent Variable: Women SI)				
95% CI				
	B	*p*	Lower	Upper
(Constant)	1.84	0.431	−2.76	6.45
SS	0.01	0.812	−0.06	0.08
ES	0.02	0.470	−0.04	0.08
FS	−0.08	0.050	−0.15	0.00
PS	−0.02	0.451	−0.08	0.03
LS	−0.06	0.257	−0.17	0.05
Lon	0.01	0.517	−0.03	0.05
CIS	−0.03	0.432	−0.11	0.05
AS	0.04	0.326	−0.04	0.11
IVR	0.06	0.249	−0.04	0.16
PD	0.17	0.000	0.13	0.21
FF	0.05	0.241	−0.03	0.13

Note: SS, Social Self-Concept; ES, Emotional Self-Concept; FS, Family Self-Concept; PS, Physical Self-Concept; AS, Academic Self-Concept; LS, Life Satisfaction; Lon, Loneliness; CIS, Community Informal Support; IVR, Internet violence and Rejection; PD, Psychological distress; FF, Family Functioning.
